# Effect of Reflow Temperature on Interfacial IMC Growth in Pb-Free/Pb Mixed-Assembly Micro-Solder Joints

**DOI:** 10.3390/mi17091034

**Published:** 2026-08-29

**Authors:** Yang Xiao, Yifan Bai, Xinyuan He, Qiming Cui, Lijuan Cheng, Rui Yang, Qi Zhang, Yong Wang

**Affiliations:** 1 College of Aeronautics and Astronautics, Taiyuan University of Technology, Taiyuan 030024, China; 2 Beijing Institute of Computer Technology and Applications, Beijing 100854, China; 3 Key Laboratory of Fundamental Science for Advanced Machining, Beijing Institute of Technology, Beijing 100081, China; 4 Jinxi Axle Company Limited, Taiyuan 030027, China

**Keywords:** Pb-free/Pb mixed assembly, micro-solder joints, reflow temperature, interfacial reactions, dissolution-diffusion kinetics

## Abstract

This study examines interfacial intermetallic compound (IMC) growth in Pb-free/Pb mixed assemblies comprising 450 µm SAC305 solder balls and Cu pads printed with a 0.12 mm layer of Sn63Pb37 solder paste. Samples were reflowed at peak temperatures of 200, 220, 240, and 260 °C, with the time above 217 °C fixed at 40 s. Scanning electron microscopy, energy-dispersive X-ray spectroscopy, and electron backscatter diffraction were used to characterize the interfacial composition, morphology, layer thickness, grain size, and kernel average misorientation (KAM). The IMC layer thickened from 1.5 µm at 200 °C to 6.0 µm at 260 °C, while KAM*_ave_* increased from 0.36° to 0.59°. At 220 °C, the IMC thickness changed only slightly to 1.6 µm, accompanied by local Pb and Ag enrichment. At 240 °C, the IMC thickness increased sharply to 4.7 µm and KAM*_ave_* increased to 0.55°, together with larger Cu_6_Sn_5_ grains and localized coarsening. At 260 °C, the IMC thickness reached 6.0 µm and KAM*_ave_* reached 0.59°, while Pb-rich regions became more pronounced and the Ag content decreased markedly, indicating substantial interfacial solute redistribution and increasingly heterogeneous IMC growth. The results link the reflow temperature to changes in interfacial chemistry, and microstructure and can help define a suitable process window for Pb-free/Pb mixed assembly.

## 1. Introduction

Ball grid array (BGA) packaging is widely used in advanced electronic assemblies because it provides a high density of interconnections within a compact area. Although electronic manufacturing has largely transitioned toward Pb-free soldering, mixed SnPb/Pb-free assembly remains relevant in some high-reliability and backward-compatible applications, where Pb-free BGA components equipped with Sn-Ag-Cu solder balls may need to be assembled using an established SnPb soldering process. In such cases, SnPb solder paste is retained to maintain compatibility with the existing assembly process, resulting in a mixed-alloy joint between the Pb-free solder ball and the SnPb paste [1]. During reflow soldering, the solder melts, wets the substrate, and forms a metallurgical joint. Temperature control is more difficult in a Pb-free/Pb mixed assembly because Sn63Pb37 melts at about 183 °C, whereas SAC305 has a liquidus temperature of about 217 °C. A peak temperature below 217 °C can melt the Sn63Pb37 solder paste while leaving the original SAC305 solder ball below its nominal liquidus temperature, although reactions between the molten paste, solder ball, and Cu pad can still occur. Once the temperature exceeds 217 °C, more extensive solder interaction becomes possible, but the increased thermal input also accelerates elemental transport and interfacial IMC growth. Excessive IMC growth is undesirable because the thickness of the interfacial layer can affect the mechanical reliability of Sn-based solder joints [1]. An appropriate reflow profile must therefore provide sufficient soldering while limiting excessive interfacial reaction. 

Previous studies have shown that interfacial IMC evolution is controlled not only by reflow temperature but also by joint geometry, elemental diffusion, and the local concentration field. Zhu et al. [2] found that reducing the gap of Cu/Sn/Cu joints increased both the interfacial IMC thickness and the Cu concentration in the solder, demonstrating that miniaturization can change the dominant diffusion process and IMC growth behavior. For SAC305-based micro-joints, Qiao et al. [3] observed pronounced nonuniform Cu_6_Sn_5_ growth under a temperature gradient and attributed this behavior to the strong anisotropy of Cu diffusion in differently oriented β-Sn grains. Huang and Yang [4] further demonstrated that the Cu concentration gradient at the solder/Cu interface is a key factor governing the size-dependent interfacial reaction and showed that Cu_6_Sn_5_ growth approaches a t^1/3^ dependence when the molten solder approaches Cu saturation. Li et al. [5] reported that increasing the size of Sn-3.0Ag-0.5Cu-0.1TiO_2_ solder joints produced thicker IMC layers and larger Cu_6_Sn_5_ grains, while the corresponding growth exponents also increased with joint size. Direct in situ observations by Huang et al. [6] revealed that pre-existing Cu_6_Sn_5_ dissolved into liquid solder during heating, remained as a thin scalloped layer during the dwell stage, and re-precipitated rapidly during cooling, with the Cu concentration gradient controlling the competition between dissolution and growth. Abdelhadi and Ladani [7] showed that the growth of interfacial IMCs in Sn-3.5Ag/Cu joints cannot be described by diffusion alone and modeled the net growth by considering the combined contributions of chemical reaction, diffusion, and dissolution. Importantly, similar interfacial effects also occur in Pb-containing systems: Yang et al. [8] found that Cu_6_Sn_5_ formed at the Sn37Pb/Cu interface exhibited pronounced crystallographic texture, and that differences in Sn diffusion along different Cu_6_Sn_5_ orientations significantly affected subsequent IMC growth. At the Cu_3_Sn stage, molecular-dynamics calculations by Gao and Qu [9] showed that Cu is the dominant diffusing species, with a diffusivity approximately seven times that of Sn at 423 K, providing an atomic-scale explanation for strongly asymmetric Cu-Sn interdiffusion. Studies on Sn/Ni interfaces further showed that the crystallographic state of the substrate can change both the interfacial IMC phase and the rate-controlling mechanism, with Ni_3_Sn_4_ growth being diffusion-controlled whereas NiSn_4_ growth is more strongly controlled by the interfacial reaction rate [10]. For Ag-containing solder systems, Ouyang and Su [11] demonstrated that a temperature gradient drove Ag transport from the hot side toward the cold side and consequently produced faster Ag_3_Sn growth at the colder interface.

Collectively, these studies demonstrate that IMC evolution is governed by the coupling of temperature, concentration gradients, crystallographic orientation, elemental diffusion, interfacial reaction, and dissolution. However, most previous investigations have considered single-solder systems or relatively simple binary interfaces. In Pb-free/Pb mixed assemblies, SAC305 solder balls and SnPb solder paste experience different melting and reaction behaviors within the same reflow cycle, so the temperature-dependent coupling among Cu-Sn IMC growth, dissolution, Pb/Ag redistribution, and local crystallographic evolution remains insufficiently understood.

In this study, Pb-free/Pb mixed-assembly micro-solder joints comprising SAC305 solder balls and Sn63Pb37 solder paste were reflowed at peak temperatures of 200, 220, 240, and 260 °C. The effects of peak reflow temperature on interfacial composition, IMC morphology and thickness, Cu_6_Sn_5_ grain size, and local crystallographic misorientation were systematically investigated using SEM, EDX, and EBSD. The aim was to clarify the temperature-dependent evolution of the interfacial microstructure and the coupled roles of IMC growth, dissolution, and elemental redistribution in the mixed-assembly system.

## 2. Materials and Methods

### 2.1. Materials and Reflow Process

**Figure 1:**
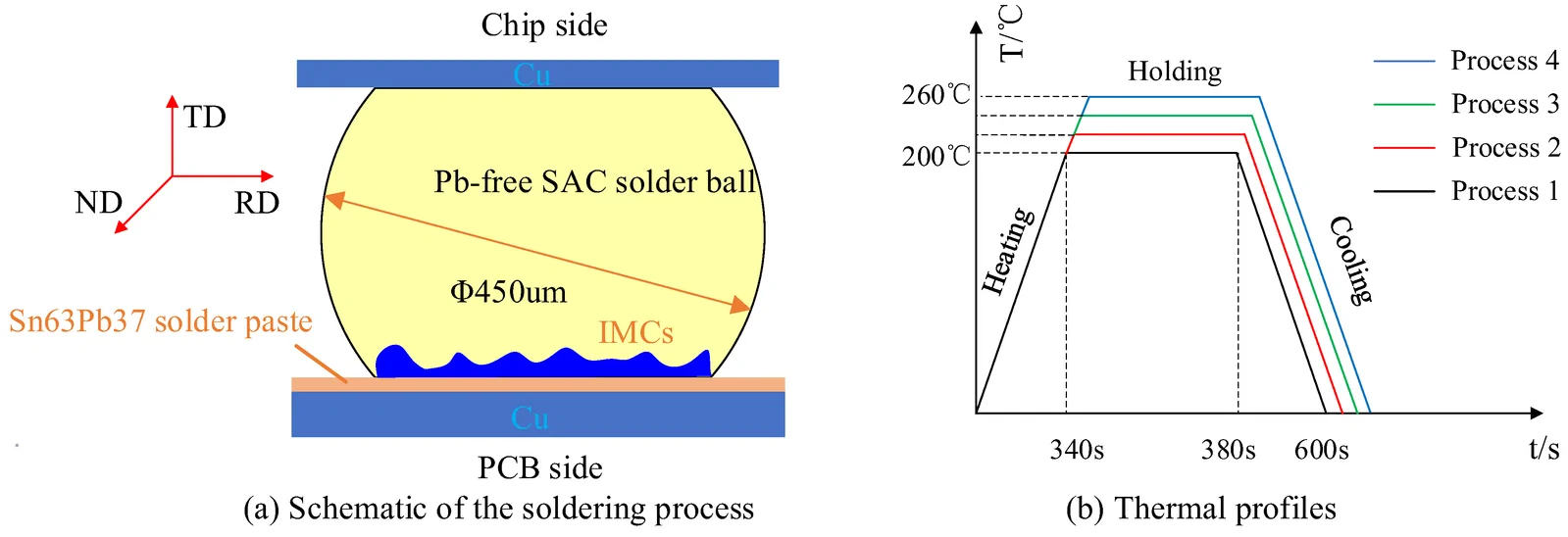
Pb-free/Pb mixed reflow-assembly process used in this study: (**a**) schematic of the soldering process, with RD, TD, and ND indicating the specimen reference directions; (**b**) thermal profiles at different peak reflow temperatures.

The materials used in the mixed-assembly joints are summarized in Table 1. Figure 1a shows the Pb-free/Pb mixed assembly used in this study. A 450 µm Sn-3.0Ag-0.5Cu (SAC305) solder ball (Indium Corporation (Suzhou) Co., Ltd., Suzhou, China) was placed between a pure-Cu chip substrate (99.95 wt.%) and a hot-air-solder-leveled Cu pad (99.95 wt.%) on the PCB. Before assembly, the pad was printed with a 0.12 mm layer of Sn63Pb37 solder paste (Indium Corporation (Suzhou) Co., Ltd., Suzhou, China). Samples were reflowed at peak temperatures of 200, 220, 240, and 260 °C. The time above 217 °C was 40 s, after which the samples were air cooled. The reflow-profile design was established with reference to IPC-7530A, Guidelines for Temperature Profiling for Mass Soldering Processes (Reflow and Wave).

### 2.2. Specimen Preparation and Data Analysis

After reflow, the solder joints were cold mounted and prepared along their maximum cross-sections. The specimens were ground with abrasive papers, mechanically polished, and then vibratory polished to obtain surfaces suitable for SEM and EBSD examination. Interfacial morphology and elemental distribution were examined using a Zeiss Supra 55 field-emission scanning electron microscope (Carl Zeiss Microscopy GmbH, Oberkochen, Germany) equipped with an energy-dispersive X-ray spectroscopy system (Oxford Instruments NanoAnalysis, High Wycombe, UK). EBSD data were acquired using a JSM-7900F scanning electron microscope (JEOL Ltd., Akishima, Tokyo, Japan) and processed in OIM Analysis 8.0 (EDAX Inc., Mahwah, NJ, USA) to examine phase distribution, grain size, crystallographic orientation, IMC-layer thickness, and KAM.

Because the interfacial IMC layer exhibited a wavy/scalloped morphology, its thickness was determined using an area-equivalent average method rather than by measuring a single local thickness. In each EBSD phase map, a fixed region of interest with a width of 100 μm and a height of 18 μm was selected along the solder/Cu interface. The area fraction of the Cu_6_Sn_5_ phase within this region was measured, and the equivalent average IMC thickness was calculated as:

(1) $$T_{IMC}=f_{{Cu}_{6}{Sn}_{5}}\times H=\frac{A_{{Cu}_{6}{Sn}_{5}}}{L}$$

where *T*_IMC_ is the area-equivalent average IMC thickness, *f*_Cu6Sn5_ is the area fraction of Cu_6_Sn_5_, *H* is the height of the selected region, *A*_Cu6Sn5_ is the cross-sectional area of Cu_6_Sn_5_, and L is the projected interfacial length. This area-based method accounts for the entire Cu_6_Sn_5_ area within the selected region and reduces the influence of local peaks and valleys caused by the wavy morphology. For each reflow condition, the reported IMC thickness represents the area-equivalent value obtained from the analyzed 100 μm × 18 μm EBSD field. The grain-size and KAM results are presented as distributions within the corresponding EBSD field, and GS_ave_ and KAM*_ave_* denote the field-averaged values of the analyzed region. Because independent replicate EBSD datasets were not available for the present analysis, between-specimen standard deviations and error bars are not reported. Accordingly, the quantitative values are used for descriptive comparison among the reflow conditions rather than for statistical significance testing.

## 3. Results

**Figure 2:**
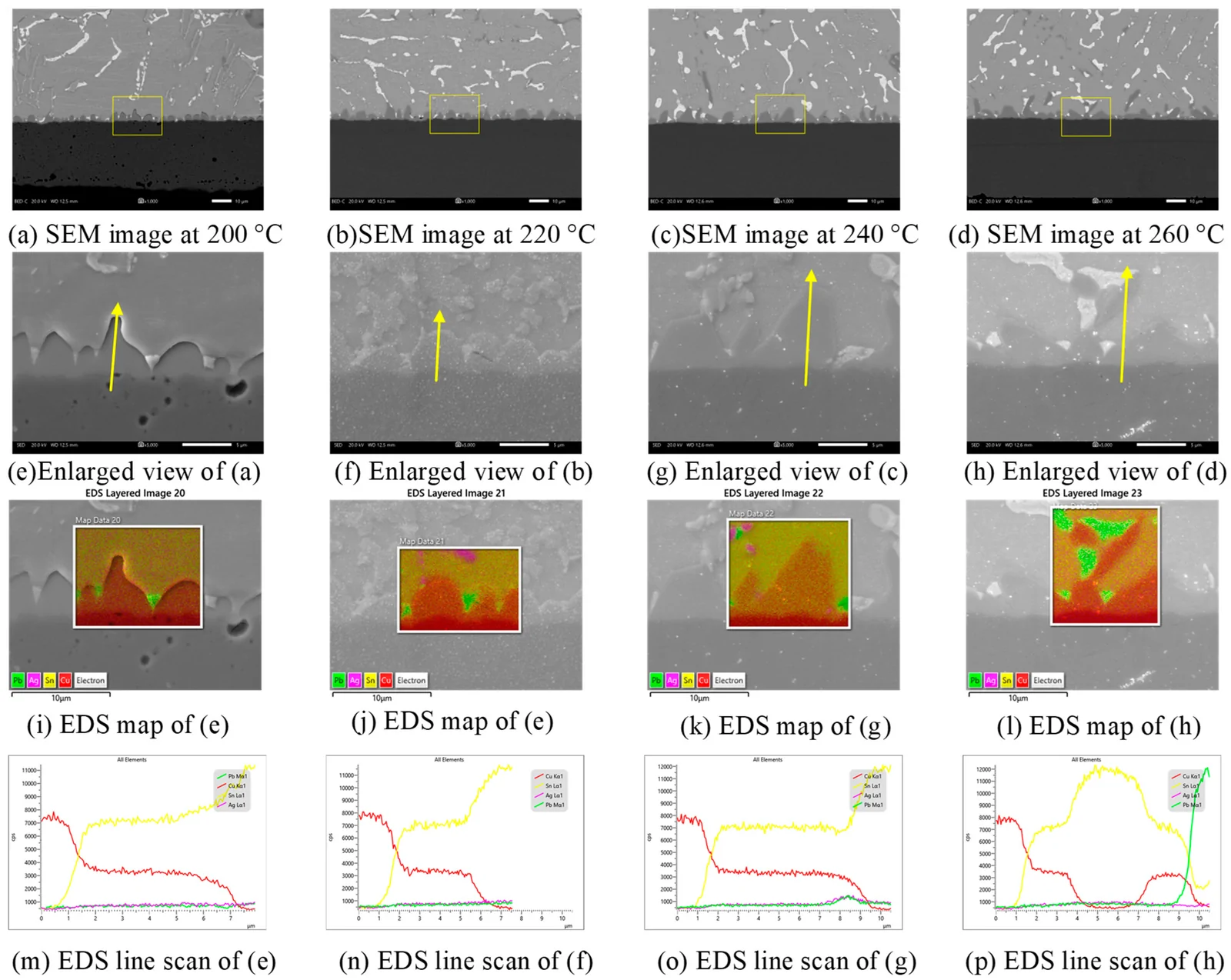
Interfacial microstructures and EDS analyses of the micro-solder joints reflowed at peak temperatures of 200, 220, 240, and 260 °C: (**a**–**d**) SEM images; (**e**–**h**) enlarged interfacial views; (**i**–**l**) regions selected for quantitative EDS analysis; and (**m**–**p**) EDS line-scan profiles. The four conditions correspond to 200, 220, 240, and 260 °C, respectively. The yellow arrows in (**e**–**h**) indicate the EDS line-scan directions, and the colors in (**i**–**l**) represent the elemental signals identified in the corresponding legends.

**Figure 3:**
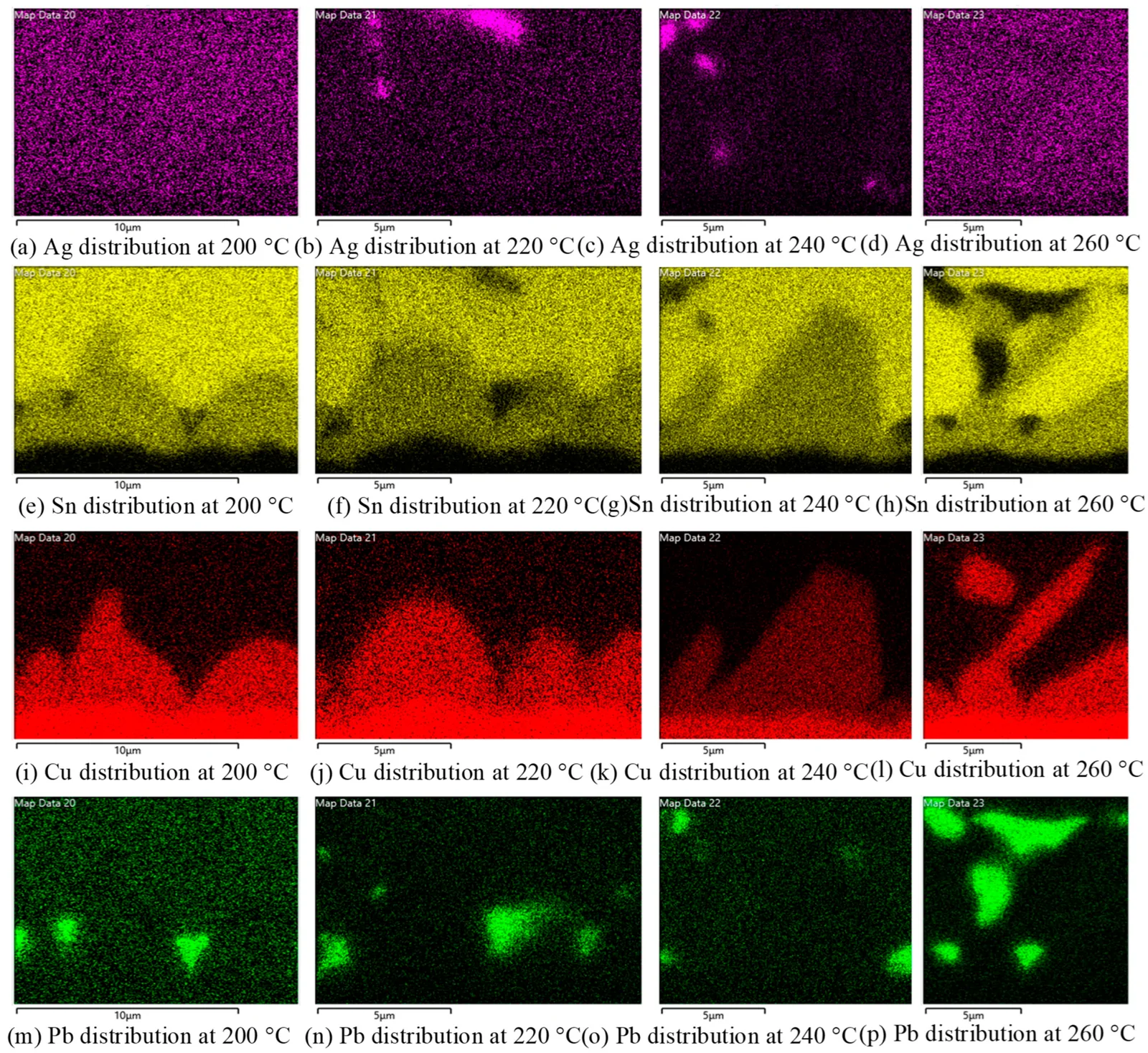
EDS elemental-distribution maps of the micro-solder-joint interfaces at different peak reflow temperatures: (**a**–**d**) Ag, (**e**–**h**) Sn, (**i**–**l**) Cu, and (**m**–**p**) Pb. Within each group, the four images correspond to 200, 220, 240, and 260 °C, respectively.

Figure 2 shows the interfaces formed between the SAC305 solder balls and the Cu pads coated with 0.12 mm of Sn63Pb37 solder paste. The colored regions in Figure 2i–l indicate the locations selected for EDS analysis. Figure 3 presents elemental maps of the regions marked in Figure 2i–l. The EDX results show that the interfacial composition changed systematically with reflow temperature. At 200 °C, the IMC layer contained 26.14 wt.% Cu (Figure 3i), 73.74 wt.% Sn (Figure 3e), and only 0.12 wt.% Ag (Figure 3a). The corresponding atomic fractions were 83.2 at.% Sn and 14.7 at.% Cu. The Cu and Sn plateaus in Figure 2m–p were used to identify Cu_6_Sn_5_ as the principal interfacial phase. At 220 °C, the Cu content increased to 27.43 wt.% (Figure 3j), while the Sn content decreased to 69.17 wt.% (Figure 3f). Local Ag and Pb enrichment reached 1.60 wt.% (Figure 3b) and 1.80 wt.% (Figure 3n), respectively. Ag accounted for 0.8 at.% of the mapped region. Compared with the layer formed at 200 °C, the IMC layer at 220 °C was less uniform (Figure 2j). Pb and Ag segregation at grain boundaries probably reduced the interfacial energy through Gibbs adsorption and restricted uniform growth.

At 240 °C, the Cu content decreased slightly to 26.85 wt.%, the Ag content reached its maximum of 1.86 wt.%, and the Sn content increased to 71.29 wt.% (Figure 2k). In this temperature range, the Pb content fell, while Ag transport was governed by grain-boundary migration. Repeated adsorption and desorption of Ag may therefore have pinned the boundaries and altered the coarsening rate. The composition changed more sharply at 260 °C (Figure 2l): Cu decreased to 23.88 wt.%, Sn to 63.91 wt.%, and Pb increased to 12.06 wt.%. The reported Cu/Sn atomic ratio of 2.68:1 approached the 3:1 stoichiometry of Cu_3_Sn, indicating that the transformation from Cu_6_Sn_5_ to Cu_3_Sn accelerated at the highest temperature. At 260 °C, enhanced atomic transport in the molten Sn-Pb phase may have promoted Pb redistribution, resulting in micrometre-scale Pb-rich regions at interfacial defects. A previous atomic-mobility assessment of liquid Sn-Pb alloys reported an activation enthalpy of approximately 11.6 kJ mol^−1^ for Pb mobility in liquid Sn [12], supporting the relatively high mobility of Pb in the Sn-rich liquid phase. The Ag content simultaneously decreased to 0.15 wt.% at 260 °C, indicating substantial redistribution of Ag at the highest reflow temperature.

**Figure 4:**
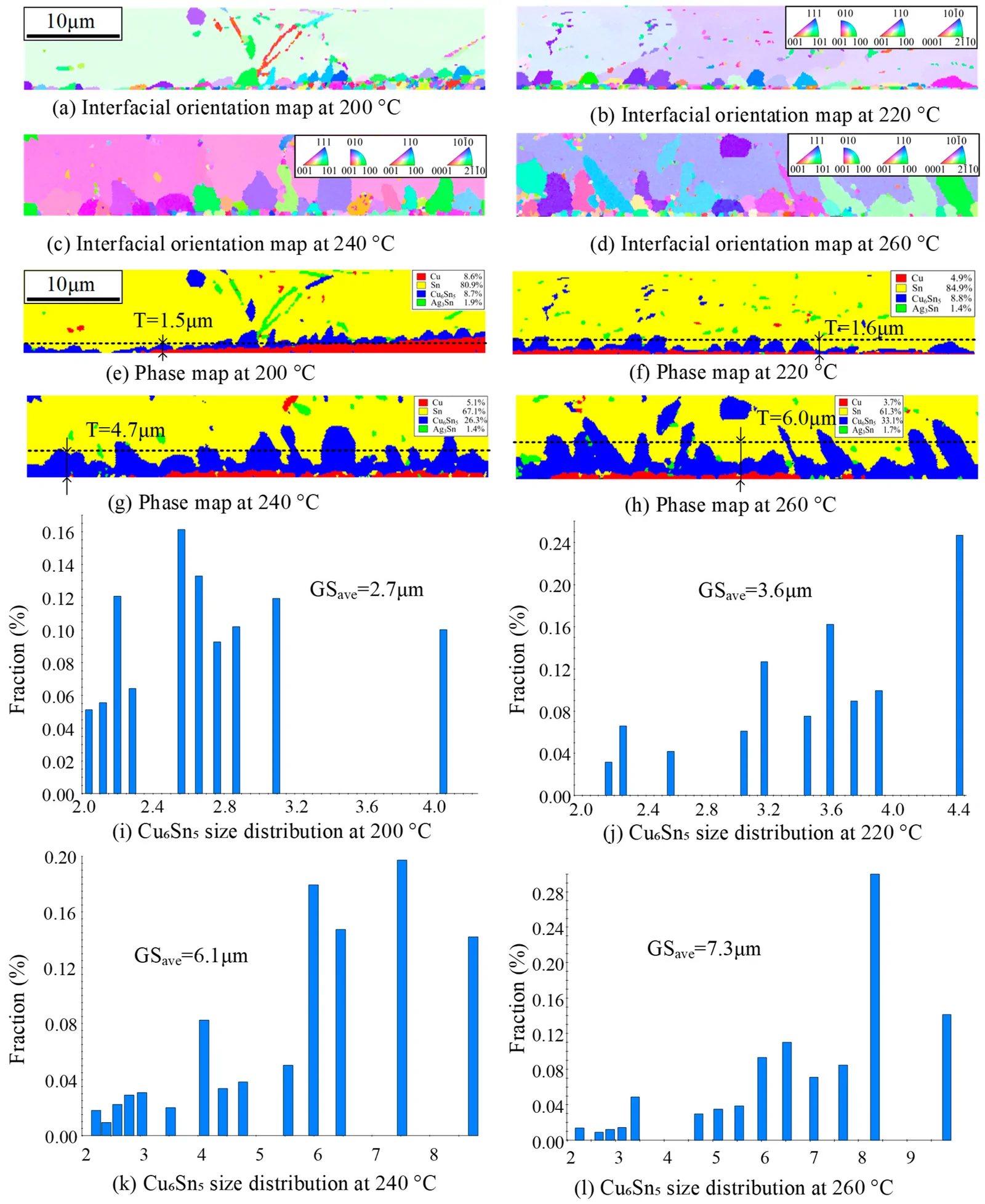
EBSD characterization of the micro-solder-joint interfaces at different peak reflow temperatures: (**a**–**d**) crystallographic orientation maps; (**e**–**h**) phase maps used for area-equivalent IMC-thickness determination; and (**i**–**l**) Cu_6_Sn_5_ grain-size distributions. Within each group, the four panels correspond to 200, 220, 240, and 260 °C, respectively.

Figure 4 presents the EBSD characterization of the interfacial microstructure under different peak reflow temperatures. The crystallographic orientation maps are shown in Figure 4a–d, the EBSD phase maps used for area-equivalent IMC-thickness calculation are shown in Figure 4e–h, and the corresponding Cu_6_Sn_5_ grain-size distributions are shown in Figure 4i–l. The average IMC thicknesses were calculated using the area-equivalent method described in Section 2.2, in which the Cu_6_Sn_5_ area fraction in a fixed interfacial region was converted into an equivalent layer thickness. This method was used to reduce the influence of local peaks and valleys caused by the wavy IMC morphology.

At 200 °C, the Cu_6_Sn_5_ grains measured 2.7 µm and the layer was 1.5 µm thick. Fine grains at the Cu_6_Sn_5_ surface indicated continued growth, while elongated Ag_3_Sn features separated some of the blocky Cu_6_Sn_5_ grains. At 220 °C, the Cu_6_Sn_5_ grains were flatter; the grain size increased to 3.6 µm, but the layer thickness changed little and reached only 1.6 µm. Pb and Ag segregation at the grain boundaries probably lowered the interfacial energy and restricted growth as the temperature increased from 200 °C (Figure 2i) to 220 °C (Figure 2j). At 240 °C, the grain size and layer thickness rose sharply to 6.1 and 4.7 µm, respectively. Grain-boundary-controlled Ag transport and the associated adsorption–desorption process may have pinned the boundaries and influenced coarsening (Figure 2k). At 260 °C, the grain size reached 7.3 µm and the layer thickness reached 6.0 µm.

**Figure 5:**
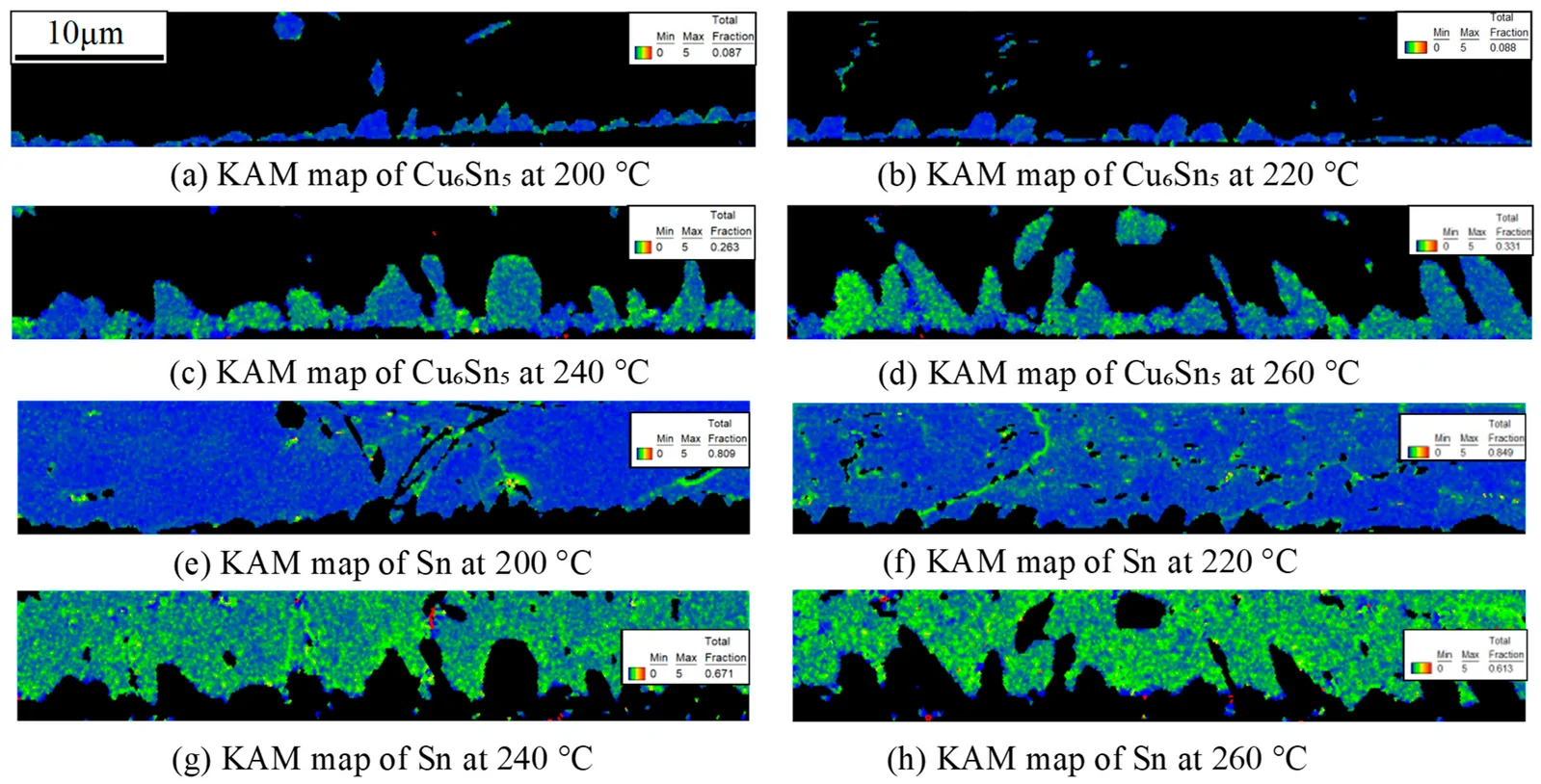

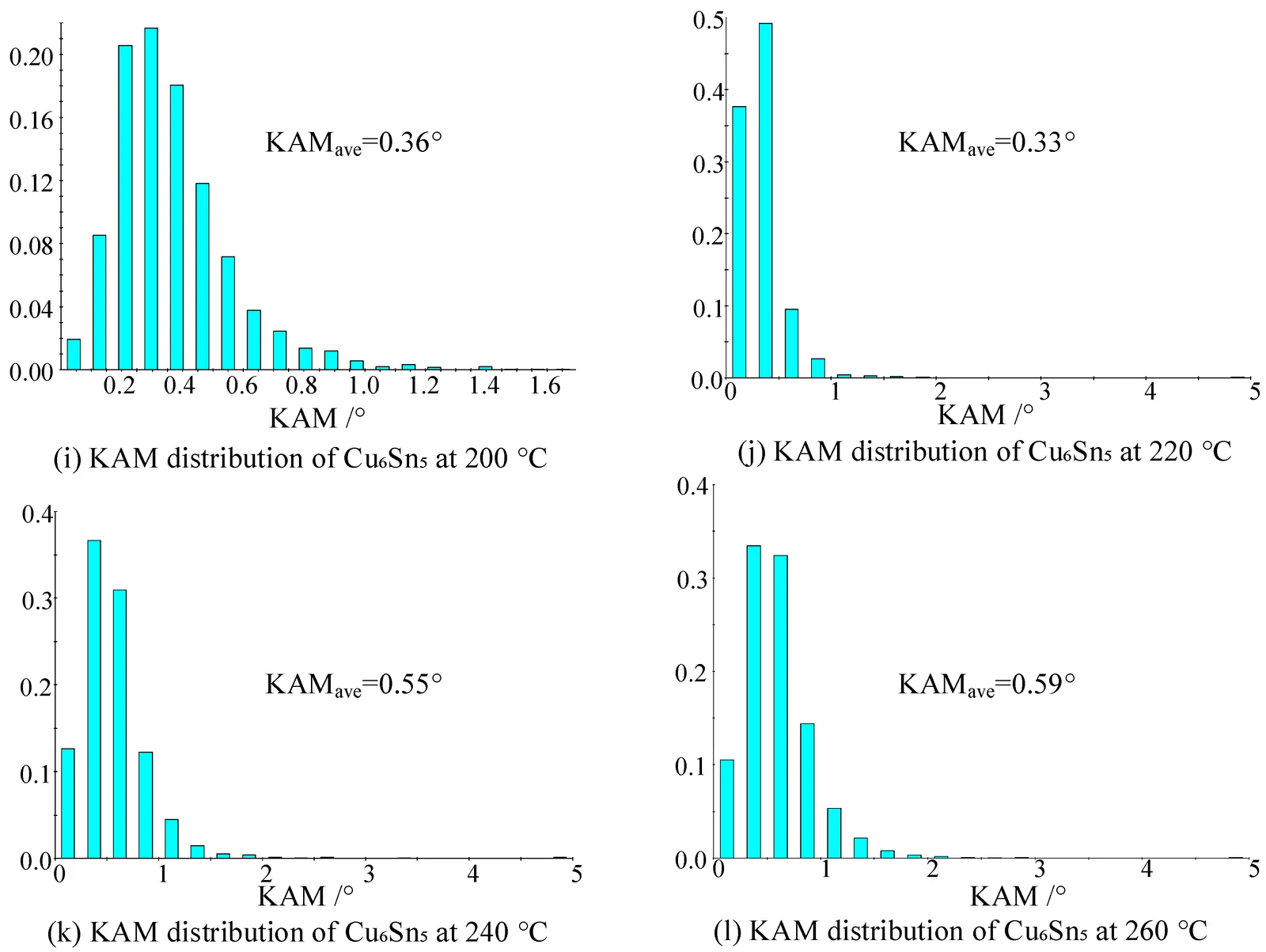
KAM characterization of the micro-solder-joint interfaces at different peak reflow temperatures: (**a**–**d**) KAM maps of Cu_6_Sn_5_ at 200, 220, 240, and 260 °C, respectively; (**e**–**h**) KAM maps of Sn at 200, 220, 240, and 260 °C, respectively; and (**i**–**l**) KAM distributions of Cu_6_Sn_5_ at 200, 220, 240, and 260 °C, respectively. KAM*_ave_* denotes the average KAM value of the analyzed Cu_6_Sn_5_ region.

The increase is consistent with faster conversion of Cu_6_Sn_5_ to Cu_3_Sn (Figure 2l), dissolution of Ag_3_Sn nanoparticles, and Kirkendall-driven redistribution. To further evaluate the local crystallographic response of the interfacial phases, kernel average misorientation (KAM) was analyzed, with KAM*_ave_* representing the average KAM value. KAM*_ave_* changed only slightly from 0.36° at 200 °C to 0.33° at 220 °C (Figure 5i,j), indicating that the local crystallographic misorientation remained broadly comparable between these two conditions. Considering the small magnitude of this difference, it was not interpreted as a significant change in local lattice distortion. When the peak reflow temperature increased to 240 °C and 260 °C, KAM*_ave_* increased to 0.55° and 0.59°, respectively (Figure 5k,l), accompanied by more pronounced high-KAM regions in the corresponding Cu_6_Sn_5_ KAM maps (Figure 5c,d). These increases indicate enhanced local crystallographic misorientation and strain heterogeneity at higher reflow temperatures and coincide with the pronounced IMC thickening, grain coarsening, and solute redistribution observed in Figure 2 and Figure 4. Therefore, KAM is used here as a supplementary indicator of the local crystallographic state rather than as a direct quantitative measure of IMC growth or phase transformation. The increase in KAM at the higher reflow temperatures is also consistent with the increasing crystallographic misorientation observed by Gu et al. [13] during thermal cycling of Sn-Ag-Cu solder joints.

## 4. Discussion

**Figure 6:**
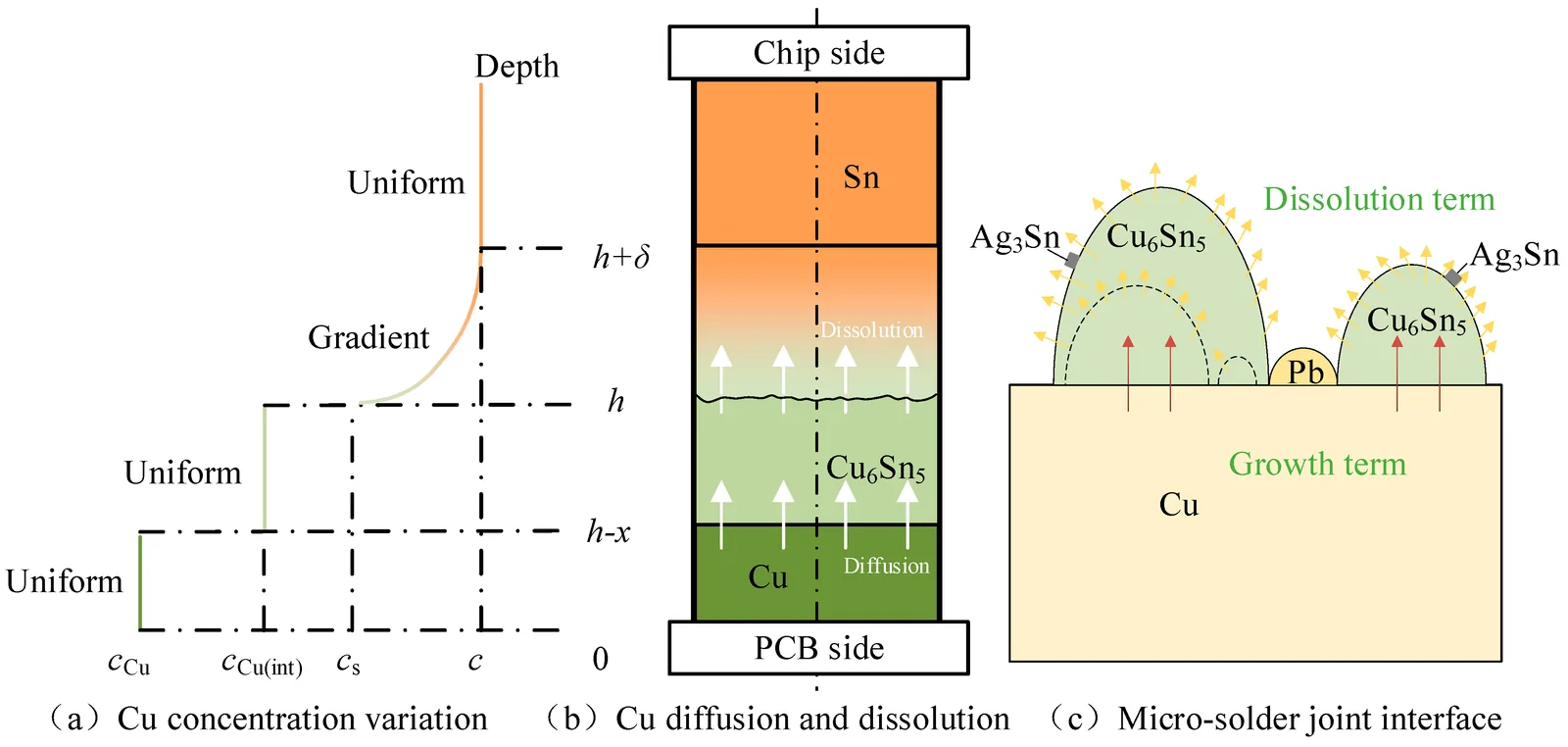
Schematic illustration of the coupled diffusion–dissolution mechanism: (**a**) Cu concentration variation across the interfacial region; (**b**) Cu diffusion and dissolution during the interfacial reaction; and (**c**) competing IMC growth and dissolution at the micro-solder-joint interface. The arrows indicate the directions of diffusion, dissolution, and interfacial growth, the colors distinguish the different phases or reaction regions as labeled in the schematic, and the dashed lines in (**a**) mark the characteristic concentration and interfacial positions.

Interfacial IMC thickness during Pb-free/Pb mixed reflow reflects the competition among growth, dissolution, and diffusion. The effect of peak reflow temperature on IMC growth was strongly nonlinear. When the peak temperature increased from 200 to 220 °C, the average IMC thickness increased only slightly from 1.5 to 1.6 µm, corresponding to an increase of approximately 6.7%. In contrast, increasing the temperature from 220 to 240 °C caused the thickness to increase sharply from 1.6 to 4.7 µm, representing an increase of approximately 194%. A further increase to 260 °C increased the thickness to 6.0 µm, approximately 28% higher than that at 240 °C. Overall, increasing the peak reflow temperature from 200 to 260 °C resulted in a fourfold increase in IMC thickness. These results indicate that the most pronounced temperature sensitivity occurred above 220 °C. At 200 °C, Cu mobility was limited and the IMC layer consisted mainly of sparse islands (Figure 2i). The layer was only 1.5 µm thick (Figure 4e), and the small, unevenly distributed grains indicate that the reaction remained near the nucleation stage (Figure 4i). In the coupled dissolution-diffusion model proposed by Dybkov [14], the growth rate, dx/dt, contains a diffusion term and a dissolution term (Figure 6c): To interpret the experimentally observed temperature dependence of IMC growth, the coupled dissolution–diffusion framework proposed by Dybkov [14] was adopted as a qualitative mechanistic framework. The corresponding Cu concentration variation and the competing diffusion–dissolution processes are schematically illustrated in Figure 6a and Figure 6b, respectively. Because only one reflow duration was examined at each peak temperature, an IMC thickness–time dataset was not available for quantitative fitting of the kinetic parameters. Therefore, Equations (2)–(4) are used to describe the competing contributions of diffusion-controlled IMC growth and interfacial dissolution rather than for numerical kinetic fitting.

(2) $$\frac{dx}{dt}=\frac{k_{1}(T)}{x}-b_{0}(T)\cdot \exp[-a(T)\cdot t]$$

where *x* is the thickness of the interfacial IMC layer, *t* is the reaction time, *T* is the absolute temperature, *k*_1_(*T*) is the temperature-dependent diffusion-controlled growth-rate constant, *b*_0_(*T*) is the initial dissolution rate, and *a*(*T*) is the dissolution-rate decay coefficient. The first term on the right-hand side represents diffusion-controlled IMC growth, whereas the second term represents interfacial dissolution. And *a*(*T*) is a temperature-dependent coefficient describing the decay of the dissolution contribution with reaction time.

The diffusion-controlled growth constant *k*_1_(*T*) is expressed as:

(3) $$k_{1}\left(T\right)=k_{0}\cdot \exp(-\frac{Q_{\mathrm{IMC}}}{RT})$$

where *k*_0_ is the pre-exponential factor for diffusion-controlled IMC growth, *Q_IMC_* is the apparent activation energy for IMC growth, and *R* is the universal gas constant. *T* has the same meaning as defined in Equation (2).

The diffusion term drives IMC growth, but its contribution decreases as the layer thickens. Growth is therefore rapid at the beginning and slows with increasing thickness [8]. The dissolution term acts in the opposite direction and decays exponentially with time because liquid Sn continuously dissolves Cu from the interface. The temperature-dependent initial dissolution rate *b*_0_(*T*) is expressed as:

(4) $$b_{0}(T)=\frac{C_{s}(T)\cdot k(T)}{\rho_{IMC}}$$

where *C_s_*(*T*) is the saturation concentration of Cu in the liquid solder at temperature *T*, *k*(*T*) is the temperature-dependent dissolution-rate constant, and *ρ*_IMC_ is the density of the interfacial IMC.

At 200 °C, diffusion-controlled growth approached a steady state and strengthened the interface. Increasing the temperature to 220 °C raised Cu mobility and made it easier for Cu atoms to leave the substrate and enter the liquid Sn. The larger supply of dissolved Cu increased the driving force for IMC formation. Because a single reflow cycle lasted only 40 s, the dissolution term had not fully decayed, and dissolution and diffusion acted concurrently. The observed Pb and Ag segregation at the grain boundaries may also have contributed to the restricted uniform growth of the IMC layer. The IMC grains therefore remained densely packed (Figure 4b), KAM*_ave_* stayed near 0.33°, and the layer thickness changed only slightly.

When the peak reflow temperature increased from 220 to 240 °C, the IMC layer thickness increased sharply from 1.6 to 4.7 µm, corresponding to an increase of approximately 194%. This pronounced thickening indicates that the interfacial reaction became substantially more temperature sensitive above 220 °C and that the balance between dissolution and growth shifted toward net IMC growth. The concurrent decrease in Pb content and redistribution of Ag may also have influenced interfacial reaction-layer evolution, consistent with the broader effects of alloying and impurities summarized by Laurila et al. [15]. Consistent with the pronounced increase in IMC thickness, KAM*_ave_* increased to 0.55°, and localized grain coarsening became evident (Figure 4c). The Cu_6_Sn_5_ layer gradually changed from a relatively dense structure to a coarser polycrystalline aggregate as it thickened. The higher reflow temperature enhances atomic mobility across the solder/Cu interface and promotes interdiffusion between the Sn matrix and Cu substrate, as schematically illustrated in Figure 6c. According to the dissolution-diffusion framework described above, the enhanced diffusion contribution at 240 °C provides a reasonable explanation for the accelerated net growth of the interfacial IMC layer.

When the peak reflow temperature further increased from 240 to 260 °C, the IMC layer thickness increased from 4.7 to 6.0 µm, corresponding to a further increase of approximately 28%. Although this increase was less pronounced than that observed between 220 and 240 °C, the continued thickening confirms that net IMC growth remained dominant at the highest reflow temperature. At 260 °C, enhanced atomic transport was accompanied by pronounced redistribution of the interfacial elements. Pb-rich regions became more evident at interfacial defects, while the Ag content decreased markedly (Figure 2l). Temperature-gradient-driven Ag transport has also been reported in microscale Pb-free solder alloys [11]. The observed solute redistribution, together with enhanced diffusion at the higher temperature, provides a possible explanation for the discontinuous and nonuniform interfacial morphology. Meanwhile, KAM*_ave_* increased further to 0.59° (Figure 5l), while the IMC layer reached its maximum measured thickness of 6.0 µm. Therefore, the results at 260 °C indicate that continued enhancement of atomic diffusion, together with solute redistribution at the interface, promoted further IMC growth and increased the heterogeneity of the interfacial microstructure. Related isothermal-aging studies of Sn37Pb/Cu joints have likewise reported continued interfacial IMC evolution with thermal exposure [16].

## 5. Conclusions

(1) With the time above 217 °C fixed at 40 s, the interfacial IMC layer thickened from 1.5 µm at a peak temperature of 200 °C to 6.0 µm at 260 °C. Over the same range, KAM*_ave_* increased from 0.36° to 0.59°. The most pronounced changes occurred between 220 and 240 °C.
(2) At 220 °C, Cu mobility increased, while Pb and Ag segregation at the grain boundaries may have restricted uniform IMC growth. The IMC layer therefore remained relatively dense, and both its thickness and KAM*_ave_* changed only slightly compared with those at 200 °C.
(3) At 240 °C, the Pb content decreased and Ag transport became more strongly associated with grain-boundary migration. The interfacial reaction shifted toward enhanced IMC growth, with the layer thickness increasing to 4.7 µm and KAM*_ave_* to 0.55°. Localized grain coarsening also became evident.
(4) At 260 °C, Pb-rich regions became more pronounced at interfacial defects, accompanied by further redistribution of Ag near the grain boundaries. These changes were associated with increasingly heterogeneous IMC growth. The IMC layer reached 6.0 µm in thickness, while KAM*_ave_* increased to 0.59°.

## Figures and Tables

**Table 1:** Nominal compositions and specifications of the materials used in the Pb-free/Pb mixed-assembly joints.

Material	Nominal Composition	Specification and Position
SAC305 solder ball	Sn-3.0Ag-0.5Cu, wt.%	Diameter: 450 µm
Sn63Pb37 solder paste	Sn-37Pb, wt.%	Printed thickness: 0.12 mm
Chip-side substrate	Cu, 99.95 wt.%	Upper side of the joint
PCB-side pad	Cu, 99.95 wt.%	Hot-air-solder-leveled surface

## Data Availability

The data presented in this study are available within the article.
